# On the mechanisms governing the critical current reduction in Nb_3_Sn Rutherford cables under transverse stress

**DOI:** 10.1038/s41598-021-86563-x

**Published:** 2021-04-01

**Authors:** Gianluca De Marzi, Bernardo Bordini, Dario Baffari

**Affiliations:** 1 Department of Fusion and Technology for Nuclear Safety and Security, ENEA, Frascati, Italy; 2 CERN, European Organization for Nuclear Research, Geneva, Switzerland

**Keywords:** Materials for devices, Applied physics, Electrical and electronic engineering, Mechanical engineering, Superconducting properties and materials

## Abstract

Within the framework of the HiLumi-LHC project, CERN is currently manufacturing 11 T dipole and quadrupole accelerator magnets using state-of-the-art Nb_3_Sn Rutherford cables. Even higher magnetic fields are considered by the Hadron Future Circular Collider (FCC-hh) design study, which plans to develop 16 T Nb_3_Sn bending dipoles. In such high-field magnets, the design pre-stress can reach considerable values (150–200 MPa) and, since Nb_3_Sn is a brittle compound, this can constitute a technological difficult challenge. Due to the significant impact that a transverse load can have on the performances of a Nb_3_Sn magnet, CERN has launched a campaign of critical current measurements of reacted and impregnated Nb_3_Sn cables subjected to transverse pressure up to about 210 MPa. In this paper, results obtained on 18-strand 10-mm-wide cable sample based on a 1-mm-diameter powder-in-tube (PIT) wire are presented. The tests were carried out on a 2-m-long sample by using the FReSCa test station, at T = 4.3 K and background magnetic fields up to 9.6 T. For applied pressures below ≈ 130 MPa, only reversible reductions of the critical current, *I*_*c*_, are observed. At higher pressures, a permanent *I*_*c*_ reduction occurs; such irreversible behaviour is due to the residual stresses generated by the plastic deformations of the copper stabilizer. This type of current reduction, whether reversible or not, is fully governed by the strain-induced variations of the upper critical field, *B*_*c2*_. At higher pressures, estimated between 180 and 210 MPa, it is indeed plausible to believe that cracking of filaments occurs, with detrimental consequences for the Nb_3_Sn cable’s electrical performances. The complete set of critical current data here presented, collected at different pressures and as a function of the applied magnetic field, allows for the first time to investigate the evolution of superconducting parameters such as the upper critical field *B*_*c2*_ in the irreversibility region, where both the effects of Cu matrix plasticization and/or cracking of filaments may occur. The experimental approach and data interpretation have a general value and can be applied to any typology of Rutherford cable.

## Introduction

The Hadron Future Circular Collider Study (FCC-hh)^1^ is developing designs for a higher performance particle collider to extend the scientific research currently being conducted at the Large Hadron Collider (LHC) and its High-Luminosity Upgrade (HL-LHC)^2,3^. The goal of the FCC-hh is to push the energy/intensity frontiers of particle colliders, with the aim of reaching collision energies of 100 TeV, in the search for new physics. The exceptional requirements of both FCC-hh and HC-LHC research studies can be fulfilled by employing higher magnetic fields and/or larger magnet apertures. In this context, Nb_3_Sn is widely accepted as the *enabling* *technology* for the construction of next generation accelerator magnets, capable of generating magnetic fields larger than 10 T. CERN is currently manufacturing 11 T dipoles^4^ and quadrupoles^5^ using state-of-the-art Nb_3_Sn Rutherford cables. Even higher magnetic fields are foreseen by the FCC-hh design study, which plans to develop 16 T Nb_3_Sn bending dipoles^6^.

The exceptional requirements set by these innovative technologies/research developments make the Nb_3_Sn superconducting coils prone to large mechanical loads due to the strong Lorentz forces acting on the coils at operating conditions.

The main purpose of the mechanical structure of a superconducting magnet is to keep the coils in compression up to the operating conditions when the maximum electromagnetic load is developed on the magnet windings. Losing pre-compression during the excitation of the magnet would cause too large deformations of the coils, which in turn could favor the movement of the strands in the coil and therefore an excessive release of heat due to friction, with subsequent quench of the magnet.

However, too high pre-compression would overstrain the conductor and therefore would limit the performance of the magnet. In facts, both the high brittleness of the Nb_3_Sn and the strong dependence of its critical current on the strain constitute a technological difficult challenge. There exists a vast literature showing that mechanical strains^7–29^ negatively affect the critical current, *I*_*c*_, of the Nb_3_Sn multifilamentary composites (or, equivalently, its critical current density *J*_*c*_). Such reduction, which is reversible below certain strain limits, depends upon both axial and transverse loads^14,16,30–32^. Current reduction has also been clearly observed in Rutherford cables^15,33,34^.

In order to evaluate the performance of each magnet structure, and based on the experience of already built magnets, particular attention is paid to a given matrix of parameters: the maximum transversal and equivalent stress during the assembly, cool down, and energization of the magnet; the loss of pre-compression on the conductor during its energization; the overstraining of the conductor and the concomitant reversible reduction of the critical current. In the various mechanical designs, it is possible to vary these parameters in an interdependent manner, being careful not to exceed the targeted maximum limits in any of the assembly/cooling/operation phases of the magnet. Regarding HL-LHC and FCC-hh, the maximum equivalent stresses are currently set to ~ 150 MPa (DS11T, MQXF)^35^ or even higher values (FCC-hh magnets)^36,37^.

Due to the significant impact that transverse pressures can have on a Nb_3_Sn magnet’s performance, in 2013 CERN decided to launch several campaigns of *I*_*c*_ measurements of Rutherford cables. To this aim, CERN has developed a dedicated sample holder^33,38^ that can measure the cables’ *I*_*c*_ under transverse loads of up to 2 × 10^6^ N/m over a length of 70 cm, in high magnetic fields (≤ 10 T) in the FReSCa test station^39^. The stress conditions reproduced by this sample holder, which can house up to 1.8 m long and 20 mm wide cables, can be considered very representative of those experienced by cables in real accelerator magnets.

Using different experimental techniques, nowadays there are several laboratories that have already carried out dedicated experiments to study the effects of transverse loads on both Rutherford wires and/or cables^14,15,30,33,40–48^. In particular, it has recently been shown^15,33,42^ that Nb_3_Sn Rutherford cables experience significant reversible *I*_*c*_ reduction already at ~ 150 MPa. Experiments on individual wires confirm these results^30^.

Although great efforts have been made to study the strain sensitivity of Nb_3_Sn wires and cables, few attempts have been made to examining in detail the *irreversible* *region*.

The irreversible region is defined as the range of stress where the critical current, after a loading and subsequent unloading, does not recover to its initial value.

Irreversibility arises from two distinct phenomena. At sufficiently high pressures, the copper of the multifilament composite—being particularly soft and ductile—tends to deform plastically. Such plastic deformations introduce a residual strain within the Nb_3_sn filaments. Furthermore, since Nb_3_Sn is a very brittle material, fractures in the filaments can also occur at sufficiently high pressures. Both these two phenomena (cracks and plastic deformations) are irreversible, and the effects on the critical current are similar, i.e. a reduction of the critical current of the wire. Consequently, in general it is not straightforward to discriminate which is the origin of the irreversible *I*_*c*_ reduction.

The main purpose of this work is therefore to establish an experimental method that allows to study in detail the irreversible region, with the aim to define a general method to clearly identify the origin of the *I*_*c*_ irreversibility in Rutherford cables.

In particular, general methods are envisaged in order: (1) to separate the irreversible and the reversible components from the overall *I*_*c*_ reduction; (2) to develop analytical methods to discriminate the origin of the irreversible component, i.e., whether the permanent *I*_*c*_ reduction is due to mechanical plasticization or to other extrinsic factors, such as filaments breakage; (3) to assess the reversible behaviour by knowing the strain state in the superconductor (decreasing of the *strain* *function*^26^ rather than breakages of the filaments); and to determine the pressure at which the cable enters in the irreversible regime.

In this paper we report and discuss the results obtained on an 18-strand 10-mm-wide cable sample based on a 1-mm-diameter powder-in-tube (PIT) wire; in addition, we propose a general method which helps to discriminate between the reversible *I*_*c*_ reduction and the *I*_*c*_ degradation, as well as between mechanical plasticization and filament breakage.

## Results

**Figure 1:**
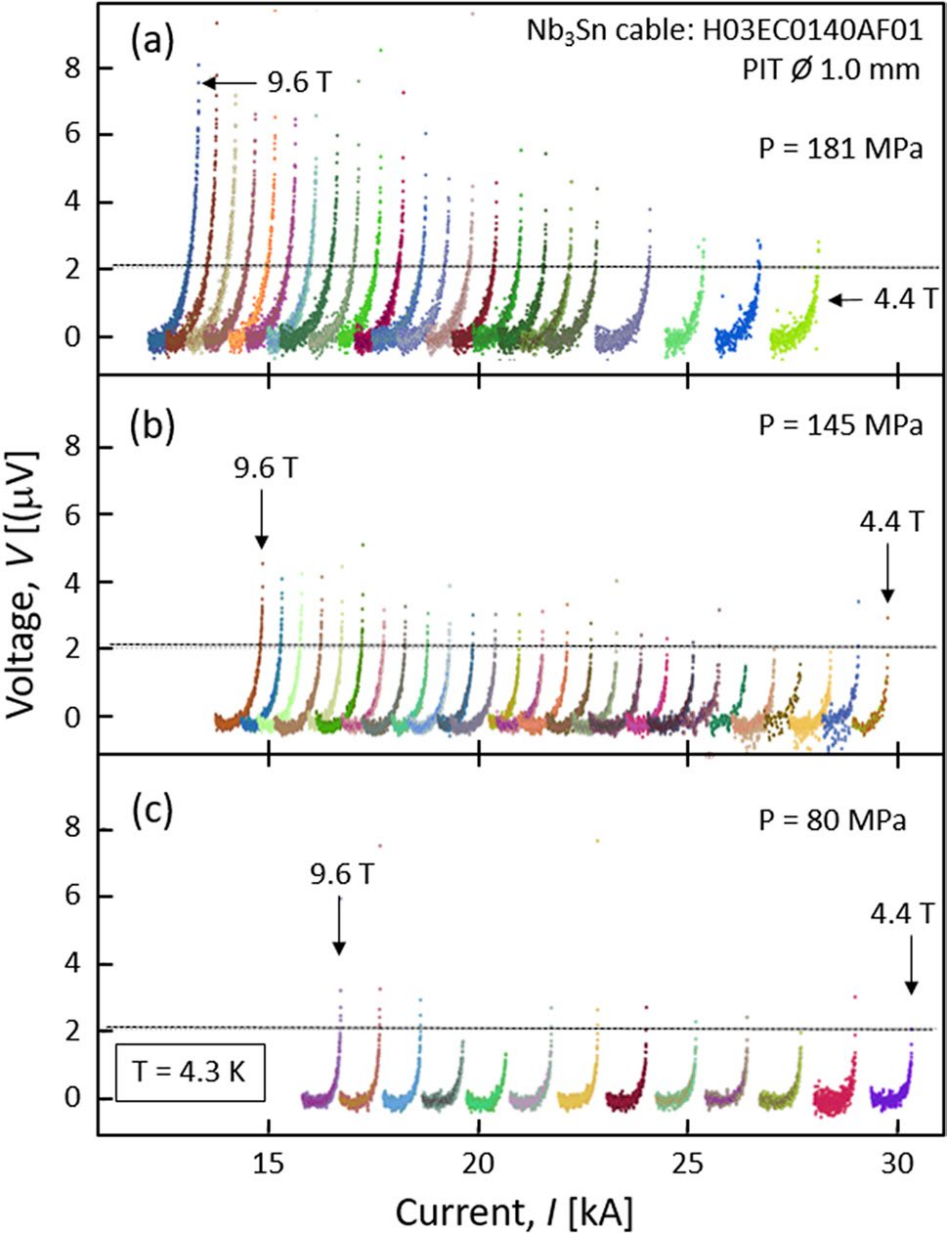
*V–I* characteristics of the PIT cable at 4.3 K collected at different background fields, at the following pressure: (**a**) 181 MPa (Test #9); (**b**) 145 MPa (Test #6); (**c**) 80 MPa (Test #1). The voltage corresponding to the chosen electric field criterion, *E*_*c*_ = 3 × 10^–2^ μV/cm, is represented by a horizontal dashed line (2.085 μV; voltage tap pair: FM).

To study the behaviour of the cable’s electromechanical properties as a function of pressure, we measured the voltage-current characteristics, *V–I*, at different transverse loads, in the range comprised between 80 and 210 MPa. At a given stress level, the *V–I* curves were measured at 4.3 K between 4.4 T and 9.6 T (background magnetic field). As an example, Fig. 1 shows the *V–I* characteristics recorded at 80 MPa (Test #1), 145 MPa (Test #6), and 181 MPa (Test #9): at a given background field, the effect of a transverse load on the *V–I* characteristics is well evidenced by shifting towards lower current values.

**Figure 2:**
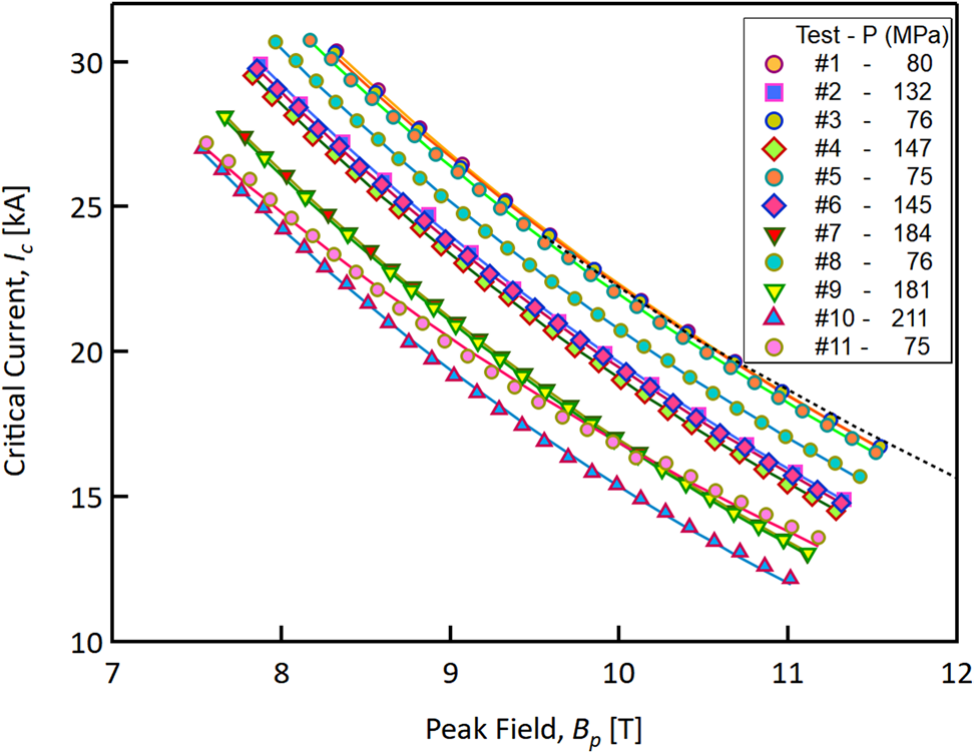
Critical current of the cable stack at T = 4.3 K as a function of the peak field, at different pressure levels ranging from 80 to 210 MPa. Markers represent the experimental data, whereas the lines are fits to Eq. (1). The black dotted line is the cable *I*_*c*_ expected from transport measurements on witness strands.

The first test (Test #1, *baseline*) was performed at the lowest applicable pressure (80 ± 3 MPa), i.e. the one resulting from the differential thermal contractions at virtually zero interference (at room temperature); at this pressure value, the critical current as a function of peak field is in line with the performances estimated from the witness sample (see Fig. 2). This latter is a strand that was extracted (before reaction) from the same cable used to prepare our sample and then undergone the heat treatment with the cable sample. The *I*_*c*_ of this witness sample was measured on a standard ITER-VAMAS type sample holder. The main test results of the witness samples are reported in Table 1. The estimated cable’s *I*_*c*_ value is obtained by multiplying the *I*_*c*_ of a witness strand by 18 (the number of strands per cable). A round witness sample, taken from the same billet used to produce the cable, was also measured showing values no more than 1% larger than the extracted witness sample. This first cable test at 80 MPa is taken as the reference *I*_*q*_ value. The following tests consisted of measuring the *V-I* curves at increasingly higher transverse pressures, up to 210 MPa. Each high-pressure test was followed by a low-pressure test at ≈ 80 MPa, with the exception of Test #6 and Test #9. Low-pressure tests were necessary to verify the onset of any irreversible behaviour in the *I*_*c*_ reduction.

**Table 1:** Main parameters of the 1.8 m-long Rutherford cable and the Nb_3_Sn PIT wire.

Parameter	Unit	Value
Copper to non-copper		1.22
Twist pitch	mm	63
Cable bare width	mm	10
Mid thickness	mm	1.81
Keystone angle	degrees	0
Number of strands		18
Strand diameter	mm	1.0
*RRR,* *virgin* *strand*		249
*RRR,* *extracted* *strand*		155
*I*_*c*_(9 T, 4.3 K)	A	1334.3
*I*_*c*_(10 T, 4.3 K)	A	1133.7
*I*_*c*_(11 T, 4.3 K)	A	968.2
*I*_*c*_(12 T, 4.3 K)	A	815.3

The critical current values refer to the measurements performed on the extracted strand (witness sample).

To evaluate the critical currents, the *V–I* characteristics were fitted to a power law function, after subtraction of both the parasitic induction offset voltage and the resistive part of the curve: *V* = *E*_*c*_ × *d*_*FM*_ × (*I*/*Ic*)^*n*^, with *I*_*c*_ and *n* free parameters, *d*_*FM*_ = 69.5 cm being the distance between points F and M (*cf*. Fig. 7).

The complete *I*_*c*_ dataset is plotted in Fig. 2 as a function of the peak field. The sequence of the tests together with the estimated pressure on the sample are reported in the legend of Fig. 2. As it can be clearly seen in the graph, the critical current is decreasing significantly with higher transverse pressures, at all applied fields.

It is important to underline that the critical currents measured during Test #1 and Test #3 are in line with the critical currents obtained from strand measurements done on witness samples tested on Ti6Al4V barrels.

In facts, the percentage difference between the expected *I*_*c*_ (from virgin wire measurements, dashed black line in Fig. 2) and the measured *I*_*c*_ is lower than ~ 1.8% at the highest applied fields. Below 11 T, the difference falls down to less than 0.5%. Similar conclusions were obtained in previous experiments ^15^.

## Discussion

The *I*_*c*_ *vs*. *B*_*p*_ curves are well described by the expression^49^:

1 $${I}_{c}\left({B}_{p}\right)=C\times {b}^{-0.5}\times {\left(1-b\right)}^{2}$$

where *C* is a constant prefactor and *b* = *B*_*p*_/*B*_*c2*_ is the reduced field. This model provides excellent fits in the region 0.2 < *b* < 0.6 for Nb_3_Sn, by only using two free parameters: the prefactor constant *C* and the upper critical field *B*_*c2*_.

**Figure 3:**
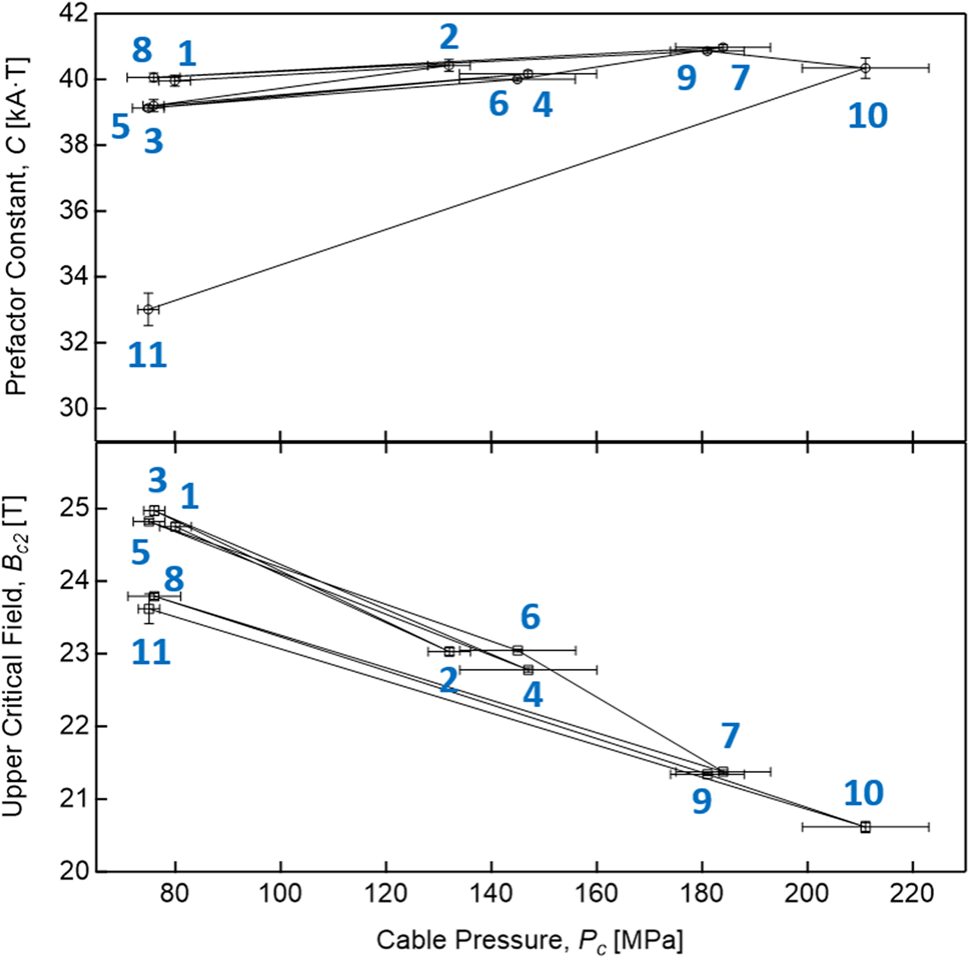
The upper critical field, *B*_*c2*_, and the prefactor constant, *C*, obtained from fits of (4) to the experimental *I*_*c*_(*B*) curves, calculated at different transversal pressures.

Equation (1) has been fitted to the *I*_*c*_ *vs*. *B*_*p*_ curves of Fig. 2 (continuous lines). The Levenberg–Marquardt algorithm is used to search for the minimum value of chi-square, defined as ${\sum }_{i}{\left[\left(y-{y}_{i}\right)/{\sigma }_{i}\right]}^{2}$, where *y* is a fitted value for a given point, *y*_*i*_ is the measured data value for the point and *σ*_*i*_ is an estimate of the standard deviation for *y*_*i*_. The obtained fitting parameters are plotted in Fig. 3. Looking at the behaviour of the fitting parameters throughout the sequence of tests, we can deduce that:

1. The prefactor *C* remains almost constant—as seen in^33^—with the exception of Test #11;
2. After an unloading test and up to Test #5, the upper critical field completely recovers its initial value (i.e., is fully *reversible*);
3. From Test #7 onwards (*P*_*c*_ = 184 ± 9 MPa), the upper critical field partially recovers but no longer returns to the baseline value (*irreversible* behaviour). In particular, after the first high-pressure test at 184 MPa (Test #7), *B*_*c2*_ reaches 96% of its initial value.

Interestingly, *C* does not practically change with pressure until Test #11. To get further insight into this finding, we start recalling the Hybrid2 variant of the Extrapolative Scaling Expression (ESE)^50,51^:

2 $${I}_{c}\left({B}_{p},T,\varepsilon \right)=\frac{{C}^{*}}{{B}_{p}}\times {\left[s\left(\varepsilon \right)\right]}^{\sigma }{h\left(t\right)}^{\frac{\eta }{2}}{b}^{p}\times {\left(1-b\right)}^{q}$$

Here, C^*^ is a constant, *h*(*t*) = (*1* − *t*^*1.5*^)$\times$ (*1* − *t*^*2*^) and *s*(*ε*) are the temperature and strain functions of the unified scaling law prefactor, *σ* is the strain function exponent parameter, *η* is an exponent parameter for *h*(*t*), *p* and *q* are the pinning-force shape parameters, and

3a $$b\equiv \frac{{B}_{p}}{{B}_{c2}\left(T,\varepsilon \right)}=\frac{{B}_{p}}{{B}_{c20}\left(1-{t}^{1.5}\right)\cdot s\left(\varepsilon \right)}$$

3b $$t\equiv \frac{T}{{T}_{c}\left(\varepsilon \right)}$$

are the reduced magnetic field and reduced temperature scaling variables, respectively.

We can simplify the ESE Hybrid2 expression by making the following assumptions:

1. The pinning-force shaping parameters *p* and *q* are fixed to the predicted values for grain boundary pinning: *p* = 0.5, *q* = 2^51^;
2. Experimentally, the exponent *σ* found in most Nb_3_Sn wires is *σ* ≈ 1.0 ± 0.3 ^7^. Here, we fix *σ* = 1^52^;
3. The exponent *η* is ≈ 2, as found in many practical multifilamentary Nb_3_Sn composites^9,50^. As in the ITER *J*_*c*_(*B*, *T*, *ε*) parameterization^52^, this parameter is fixed to *η* = 2;
4. In Eq. (3b), the strain dependence of *T*_*c*_ can be neglected, thus implying that *h*(*t*) is not depending on strain.

Based on such assumptions, and after writing C^*^ = *C′·B*_*c20*_, Eq. (2) assumes the following form^53^:

4 $${I}_{c}\left({B}_{p},T,\varepsilon \right)={C}^{^{\prime}}\frac{{B}_{c20}}{{B}_{p}}\times s\left(\varepsilon \right)\times \left(1-{t}^{1.5}\right)\times \left(1-{t}^{2}\right){\times b}^{0.5}\times {\left(1-b\right)}^{2}={C}^{^{\prime}}\times \left(1-{t}^{2}\right){\times b}^{-0.5}\times {\left(1-b\right)}^{2}$$

It can be clearly seen that the pre-factor C of Eq. (1):

5 $$C=C\mathrm{^{\prime}}\times \left(1-{t}^{2}\right)$$

is a function of only the temperature, and does not depend on the strain. Hence, it is expected that in the fitting procedure the pre-factor *C* remains practically unchanged through the tests until filament cracks occur.

Up to 184 ± 9 MPa, the reduction of both *I*_*c*_ and *B*_*c2*_ is essentially due to the strain state and not to the presence of any cracks within the superconducting filaments. This implies that the reduction is *reversible*; in facts, any *B*_*c2*_ reduction induced by a modification of the strain state is, by its nature, reversible.

However, a small amount of *I*_*c*_ *permanent* *reduction* was also observed. This *permanent* *reduction* is by definition an *irreversible* phenomenon; i.e., after a high-pressure test and subsequent unloading, the superconducting properties do not recover to their original value. The observed irreversibility comes from the residual stresses generated by the plastic deformations of the fully annealed Cu stabilizer (yield strength = 40 MPa^54^), which changes irreversibly the strain state inside the superconducting Nb_3_Sn compound. The fact that up to 184 MPa the reduction is dominated by the strain and not by any cracks is also demonstrated by the experimental observation that repeated pressure cycling at 180 MPa do not change *B*_*c2*_ and *C* significantly. Conversely, cycling loads on samples containing cracks showed a continuous build-up of degradation.

While we have not yet experimentally confirmed all sources of plastic strain induced in the filaments, in this article we assume that the remnant Cu–Sn, and other core phases, do not impose strain from the inside of the filament, and the induced strain rather arises from the stabilizing Cu surrounding the filaments/Nb barriers. To strengthen this assumption, we recall that the cores of PIT strands contain a large number of very small voids^55,56^ which are clustered in large clouds within the volume inside the tubes^57^. The morphology and number of these voids strongly impact the distribution of the mechanical stress inside the strands' cores, as well as their mechanical tolerance. In this sense, we think that any stress would just cause the core to fracture before it induces strain onto the main A15 layer.

Several works tried to express the behaviour of the critical current as a function of the 3D strain state. An exponential scaling law for the strain function was proposed by Bordini et al*.*^26^. This law was applied to describe the reversible critical current reduction^54,58^, as measured in a PIT Rutherford cable stack^33^. Considering that: (1) the aforementioned cable is identical to the one reported in this work; (2) the same experimental set-up was used; (3) the experimental data plotted in Fig. 2 are in line with previous measurements, we can assume that the evolution of *B*_*c2*_ during the test series might be also described by an exponential scale law for the strain function (up to the threshold pressure for filament cracking).

**Figure 4:**
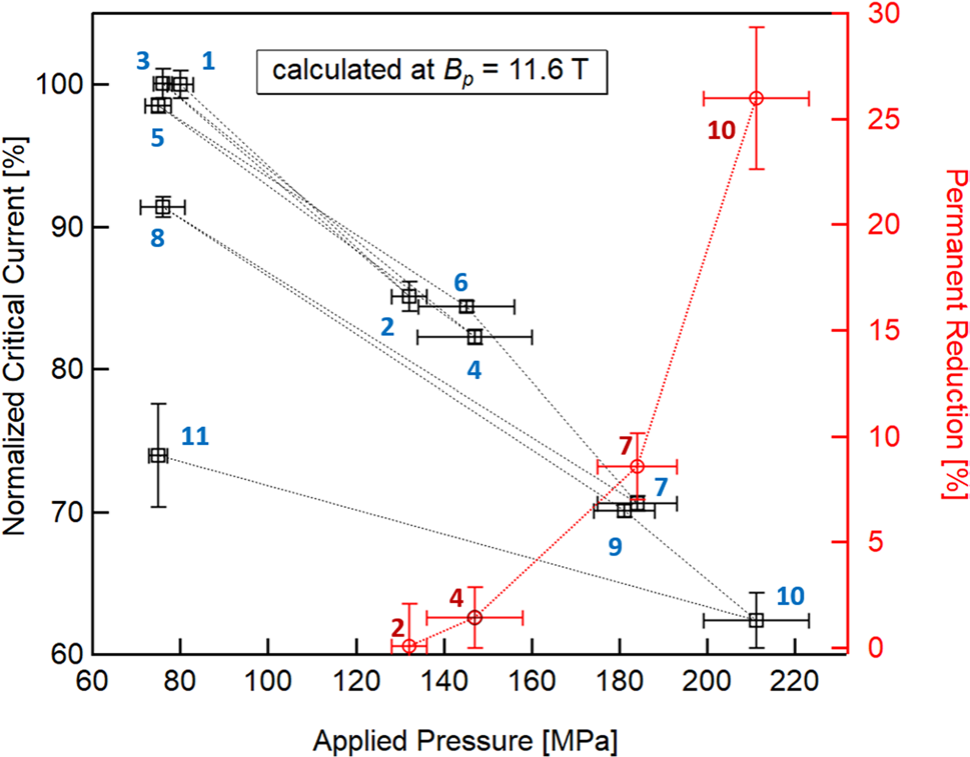
Percentage of reversible (black squares) and permanent reduction (red diamonds) of the critical current with a transversal pressure applied along the cables for a peak field of 11.6 T. The results from the first test (the *baseline* performed at 80 MPa) is used as reference for the normalization.

To quantify the irreversible effects on the superconductor performance, we can define the *I*_*c*_ *permanent* *reduction* as the percentage change between the *n*-th low-pressure test and the baseline: $100\times \left|{I}_{c}^{\#n}-{I}_{c}^{\#1}\right|/{I}_{c}^{\#1}$. This calculation can be made at any magnetic field; in this analysis, we chose *B*_*p*_ = 11.6 T, the maximum peak field available for any test performed, see Fig. 2. The results are shown in Fig. 4. For sake of clarity, the same graph also shows the critical current normalized to ${I}_{c}^{Test1}$.

At about 130 MPa, a significant reduction of the critical current is observed: the *I*_*c*_ is 86% of the baseline current. This reduction is caused by the reduction of the *B*_*c2*_ (93%, see Fig. 3) induced by the decreasing of the strain function *s*(*ε*). This idea is also supported by Test #3 at 76 MPa that showed full recovery of the critical current (> 99%). The following tests performed at higher pressures showed a further decrease of the critical current and the upper critical field. Each unloading test following a high-pressure test (Tests #5, #8, and #11) showed a progressive increase of the *I*_*c*_ permanent reduction, from 1.5% up to over 25%. The maximum pressure, 210 MPa, was applied during Test #10.

Around 180 MPa and at 11.6 T, the critical current is reduced by 30% compared to its baseline value, as well as *B*_*c2*_ (86%). These results are consistent with what was found in a previous test on a similar cable based on internal-tin rod-restacked wires (RRP)^15^, in particular the strong reduction of the *B*_*c2*_ (and consequently of the critical current) with the applied transverse load, a reduction due to a decrease of the strain function.

The subsequent last test at 75 MPa (Test #11) showed a clear jump of the permanent reduction above 25%, with partial recovery of *I*_*c*_ (< 75%). It is important to remark that *B*_*c2*_ returns to the value of the previous low-pressure test (Test #8), whereas the prefactor *C* is significantly reduced. The anomalous behaviour of *B*_*c2*_ and *C* found in Test #11 suggests that another permanent reduction source is overlapping the one originating from the plastic deformations of the fully annealed Cu stabilizer.

At very high pressure values, due to their brittleness, fracture of Nb_3_Sn filaments may occur if the mechanical loading exceeds the Nb_3_Sn crack initiation It is worth mentioning here the extensive metallographic analysis^59,60^ carried out for the ITER cable-in-conduit conductors, in order to explore the role of the Lorentz loads in producing fractures in the brittle Nb_3_Sn filaments. Detailed filament crack count and fracture analysis classification have unambiguously evidenced the presence of ratcheted crack opening under electromagnetic cycling. It has recently been shown^61^ that such cracks tend to propagate along the transport current direction (longitudinal cracks), with subsequent shrinkage of the effective superconductive cross-section. These are different from those generated by bending or tensile stress, which cause transversal cracks in sub-elements^62^.

According to our findings, it is very likely that, at 210 MPa, filaments breakage occurs. However, it is plausible that the cracks could start in a pressure window comprised between 185 MPa and 210 MPa, as the repetition of multiple tests at 180 MPa did not show any further degradation. It should be emphasized that these threshold pressure values are specific to this specific cable. The same holds for the boundary pressure above which the reversibility/plastic deformation of the copper stabilizer occurs. It is known, in facts, that cables with different degree of compaction, cables comprised of wires with different layouts, residual-resistivity-ratio (RRR), and/or different methods of production (PIT vs. RRP), as well as cables that undergo a less aggressive heat treatment^20,21^, can change these numbers significantly. However, since our cable is fully reacted, we do not expect it to become mechanically stronger with a different heat treatment.

To further, corroborate the hypothesis of filament cracking at the highest pressure, we investigated the behaviour of the *n*-value, which is inversely related to the width of the *V–I* transition, thus defining its sharpness.

**Figure 5:**
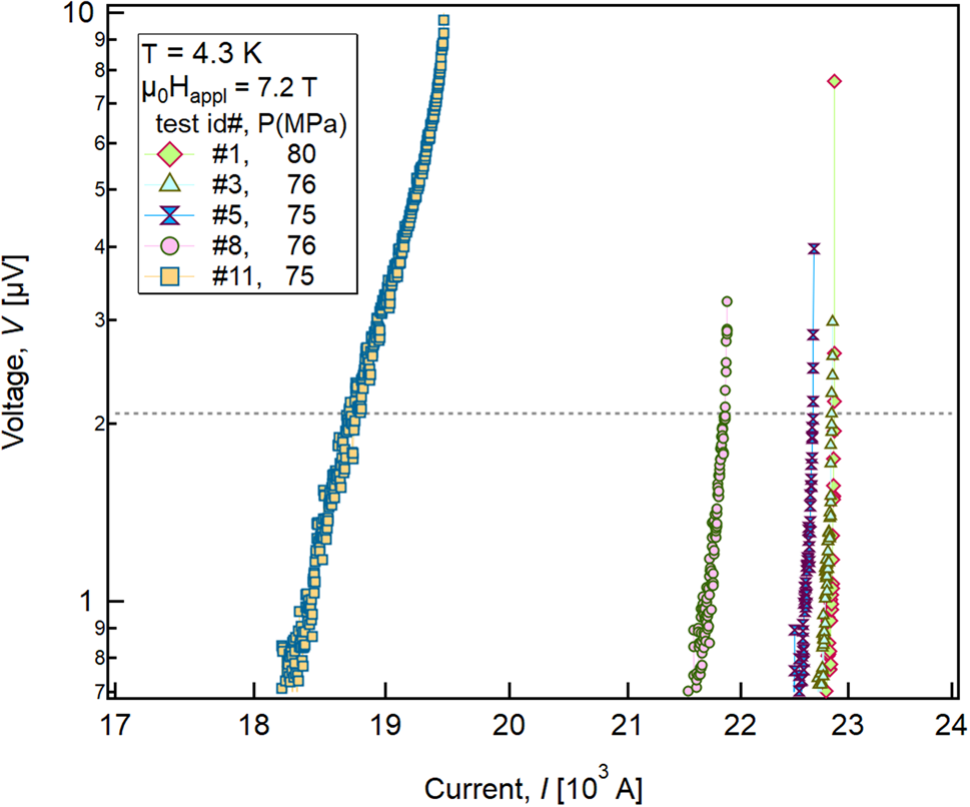
*V–I* characteristics of the PIT cable at 4.3 K with an applied magnetic field of 7.2 T for the Tests #1, #3, #5, #8 and #11 performed near 80 MPa. The *V–I* characteristics of tests #1, #3, and #5 are similar to that of Test #8, whereas smoother transitions are observed in the *V–I* curves of Test #11.

As for the critical current, the wire’s *n*-value is affected by extrinsic effects like filament non-uniformities. Due to the thermal runaway and the low number of measurement points in the resistive transition, an effective *n*-value for the cable cannot be rigorously defined. However, by comparing the *V–I* characteristics of the full set of low-pressure tests (Fig. 5), there is clear evidence of a shape change in the *V–I* curves of Test #11. This is due to the increased portion of transport current transferred to the stabilizer below critical conditions induced by the shrinkage of the effective superconductive cross-section. This effect was not observed in previous high-pressure tests (Test # 9 and Test # 10). This may be explained by the fact that the cracks did not lead to a noticeable degradation as long as the stress was retained, thus preventing any crack opening. During the final unloading test, the cracks were then free to open; however, it cannot be ruled out that the cracks increased significantly in number when a high bladder pressure was applied to remove the keys of Test #10 and return from ~ 200 MPa to 80 MPa.

As a concluding remark, we underline that the reduction of the critical current under transverse stress is a general phenomenon observed in all Rutherford cables^15,33,34^: up to a certain load the reduction is completely reversible and is due to the elastic strain, induced on the composite wire, that causes a reduction of the *B*_*c2*_^15,33^; for larger stresses, a permanent reduction appears and it is always determined by the combination of the effects of plastic deformation of the copper matrix and cracks in the Nb_3_Sn filaments, as seen for example in cables based on RRP wires^15,34^.

In particular, from the transverse pressure response in PIT and RRP cable, Gao et al. found a stress limit > 150 MPa (PIT) and > 190 MPa (RRP), when the permanent reduction of the critical current at about 12 T is *∆I*_*c,irrev.*_ = − 2% with respect to its unstressed initial value. The experimental results by Gao et al. show that both types of cable behave qualitatively in a similar way; however, in discussing the mechanisms behind the origin of *∆I*_*c,irrev.*_, the authors do not mention the role played by the plasticization of copper and by the cracks of the Nb_3_Sn filaments, nor the effects caused on *B*_*c2*_ and the prefactor *C*. On the contrary, Bordini et al. and Duvauchelle et al. indeed studied the effect of the transverse pressure on *B*_*c2*_ but focusing only on the reversibility zone, thus not proving the effect of filaments cracking on the prefactor *C*.

We have therefore demonstrated for the first time that it is possible to discriminate between the effect of Cu plastic deformations and filament cracking by analyzing the behaviour of *B*_*c2*_ and *C* vs. applied pressure. This methodology is generally applicable, and it can be used to analyze the data obtained on Rutherford cables comprised of different layouts/wires.

## Methods

### Preparation of the cable stack

**Figure 6:**
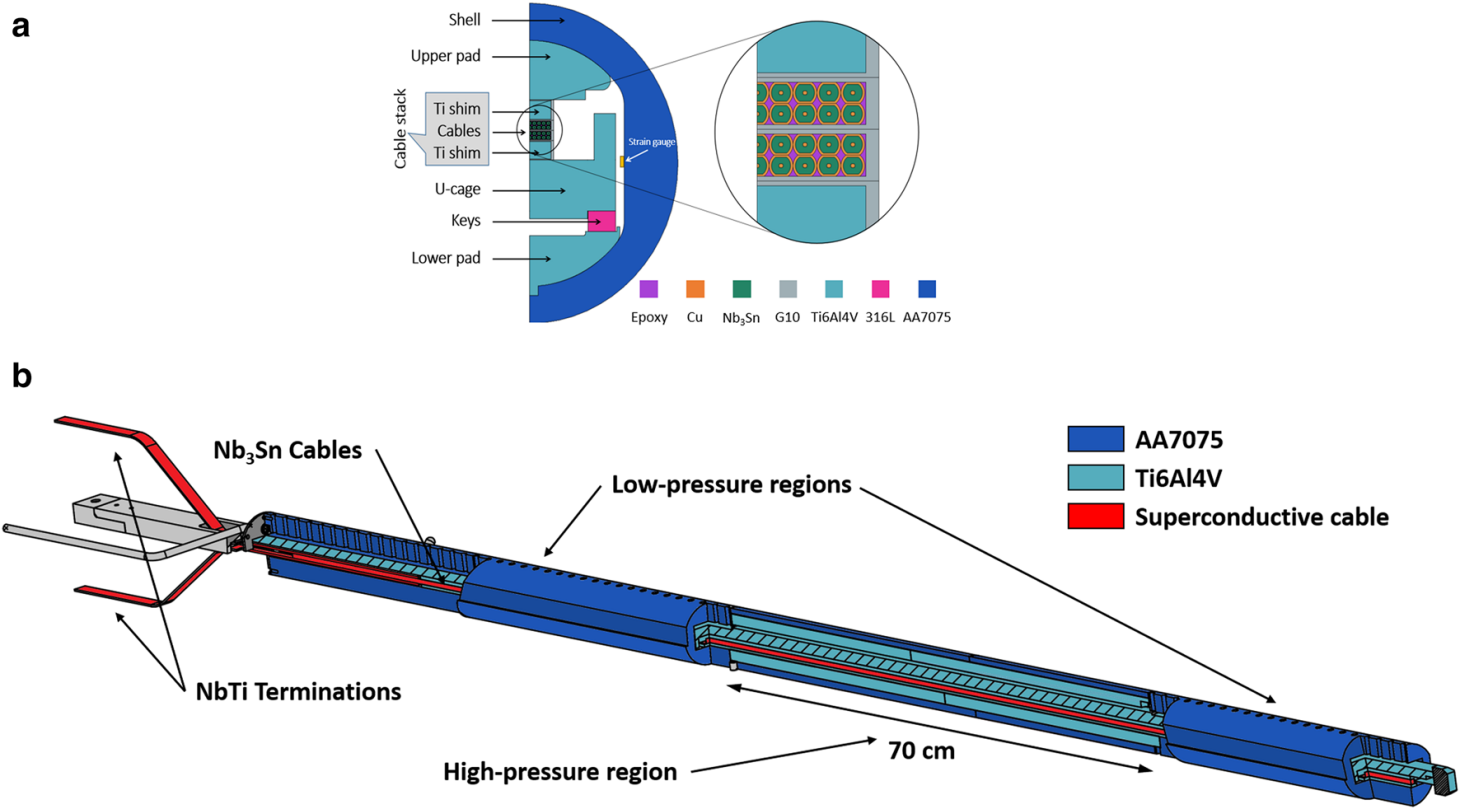
The FReSCa sample holder for critical current measurements of long cables under transversal load. The high-pressure region extends over 700 mm field uniformity length, and it is representative of the pressure that the conductor experiences in a real accelerator magnet. Top: cross-section of the high-pressure region, showing the stainless keys used to create mechanical interference; bottom: an axonometric projection of the entire assembly.

**Figure 7:**
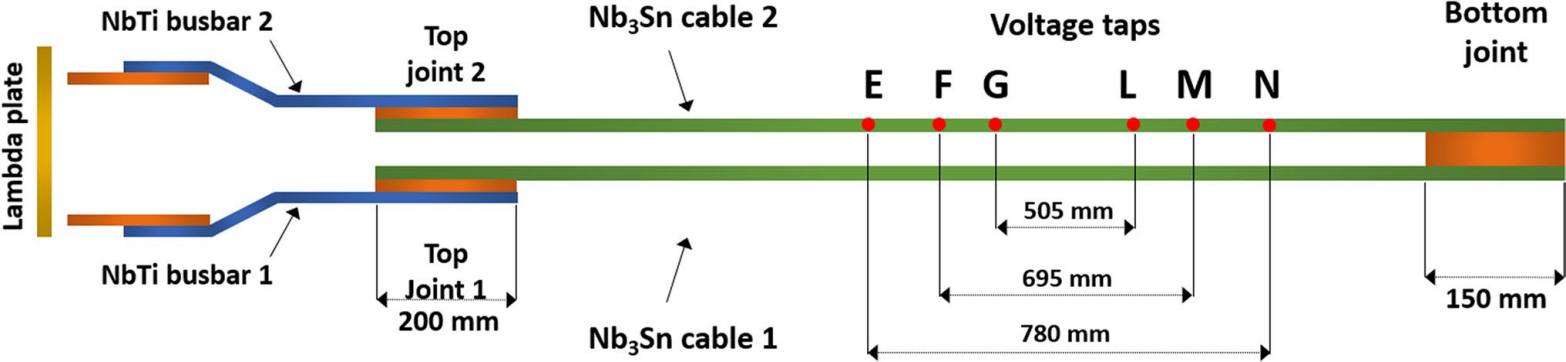
The electrical scheme used for the cable’s *I*_*c*_ measurements in the FReSCa test facility at CERN.

The sample was made of a stack of two rectangular 1.8 m long Rutherford cables based on eighteen 1 mm diameter Nb_3_Sn PIT wires^63^, which were produced from one single billet, and two 1.6 m long Ti6Al4V rectangular bars respectively 3 mm and 4 mm thick (see the 2D schematic of Fig. 6). The hairpin sample layout with the position of terminations, joints, and voltage taps as tested in FReSCa is schematically shown in Fig. 7. Each polarity is connected to NbTi busbars at the top joints. The main characteristics of the cable are summarized in Table 1. The Ti6Al4V bars on top and bottom of the superconducting cables soften any stress concentrations that could occur on the cables due to possible discontinuities in the upper and lower pads.

To ensure proper electrical insulation, each cable was wrapped twice with a 0.15 mm thick fiberglass ribbon (E-glass sized with TD22G). The two cables were then individually wrapped with a further layer of fiberglass, as well as the Ti6Al4V bars.

Finally, the entire stack, consisting of the superconducting cables in the center and the bars above and below, was wrapped with a further layer of fiberglass. The fiberglass underwent a thermal desizing process at 300 C for 72 h before use, in order to avoid any contamination during the subsequent heat treatment.

The bottom part of each cable was peeled off over a length of 20 cm, and a 0.3 mm thick layer of copper was placed between the two cables along the 20 cm length in order to ensure a better electrical interface. The bottom joint was then wrapped again with fiberglass ribbon. Several pairs of voltage taps, consisting of 1 mm × 13 mm Cu strips, are placed directly on top of the stack through the fiberglass wrapping. The two legs of the stacks are not soldered together; the integrity of the joint is in facts guaranteed by the copper diffusion bonding occurring during the subsequent reaction heat treatment.

In order to form the superconducting A15 phase, the stack was reacted according to the following heat treatment (HT): 100 h dwell at 620 °C; 120 h dwell at 640 °C with temperature ramp rates of 50 °C/h. The diffusion of Cu during HT guarantees good electrical contact between the different parts of the bottom joint and between the voltage taps and the sample. The RRR values for a virgin and an extracted strand are reported in Table 1. Such values indicate that the diffusion of Sn in the stabilizer matrix is negligible. Based on these RRR values, the *I*_*c*_ of the witness wire samples, and the type of PIT strand employed, we can reasonably assume that the irreversibility limit is located on the Strain Irreversibility Cliff (SIC) plateau^21^.

After the HT process, the sample was vacuum impregnated with epoxy resin in a dedicated impregnation mould.

The CTD101K epoxy resin was mixed following the recommended mixing procedure: (1) the resin was heated to 60 °C for approximately 1 h to liquefy the system; (2) weighted components (100 parts of resin; 90 parts of hardener; 1.5 of accelerator component) were combined into a container equipped with heating and mechanical stirring for 10 min, at 60 °C and 0.4 mbar; (3) the mix was degassed for approximately 20–40 min until bubbles stopped developing from the mixture.

Subsequently, a vacuum impregnation was carried out at 60 °C by applying the vacuum to one end of the reaction mould while maintaining the pressure at 0.4 mbar in the epoxy container connected to the other end. Finally, the resin was cured for 5 h at 110 °C, followed by a post-cure of 16 h at 125 °C. We observed no visible cracks in the resin at the end of the test series.

### Sample holder and electrical scheme

The reacted and impregnated PIT cable stack was placed in the middle of the U-cage, see Fig. 6. The impregnation was peeled off at the end opposite to the bottom joint, and the two cables were individually joint to a NbTi busbar over a length of 20 cm. The NbTi cables were connected to the current leads of the FReSCa insert.

Figure 7 shows a schematic of the electrical circuit used to measure the sample’s critical current. As discussed in detail previously, the two superconducting cables (bifilar configuration—positive and negative polarity) are connected in series via a 150 mm long, 0.3 mm thick Cu foil forming the bottom joint. Each polarity is connected to the NbTi busbars at the top joint. The resistance of the top joints was found to be ≃ 0.2 nΩ; a slightly higher value was obtained for the bottom joint ($\lesssim$ 1 nΩ). The NbTi busbar-Cu resistance was ≃ 1.5 nΩ.

The position of the voltage taps and the labels of the measured segments are also reported in Fig. 7. The segments EN, FM, and GL are 780 mm, 695 mm, and 505 mm long, respectively. The segment FM covers the homogeneous background field length of the FReSCa dipole.

### Assessment of the pressure acting on the sample

The transverse pressure is applied by means of the “*bladder* *and* *key*” method^64^. This consists of inserting, at room temperature (RT), a bladder inside the sample holder (more precisely, in the gap between the U-cage and the lower pad, as shown in Fig. 6, left side).

The bladder is then inflated with pressurized water, thus pushing the lower and upper pads towards the aluminium shell. This allows the insertion of the 316L stainless steel keys. Once the bladder is depressurized and removed, the keys maintain a certain pressure on the cable through mechanical interference: in facts, since the keys are larger than the available space given by the U-cage and the lower pad, the resulting contact forces will generate interference prestresses, distributed throughout the whole assembly. When the sample holder is cooled in the liquid helium bath, the stress due to the differential thermal contractions between the Ti6AL4V pads and the aluminium shell adds to the interference stress. Further details can be found in previous works by Bordini et al.^33,38^.

**Figure 8:**
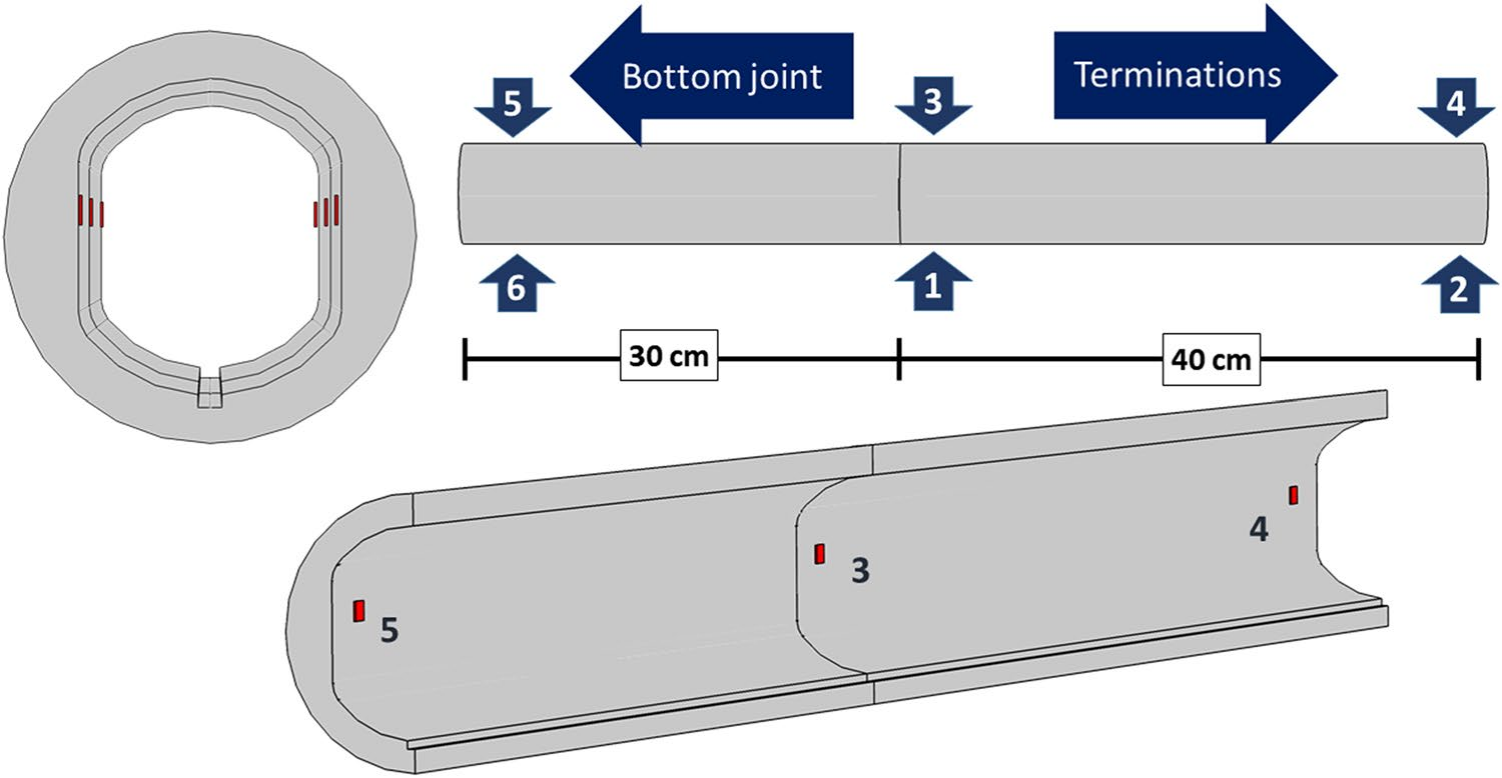
Sketches of the positions of the strain gauges on the inner walls of the Al shell. The strain gauges allow for the measurement of both the azimuthal and longitudinal components.

Twelve strain gauges were installed on the inner walls of the aluminium shell, in the positions depicted in Fig. 8. The gauges allowed measuring the strain both in the longitudinal and in the azimuthal directions. A compensation strain gauge was also placed on a small piece of aluminium outside the high-pressure region in order to evaluate the mechanical strain from the total measured strain, by a proper subtraction of the thermal strain.

The pressure acting on the superconducting cable at low pressure was estimated from the strain gauges measurements via a 3D FEM model with the commercial simulation environment Comsol MultiPhysics 5.4^65^, using the 3D *Solid* *Mechanics* interface of the *Structural* *Mechanics* module^66^. The mechanical interference was simulated using *Contact* *surface* *offset* in the Contact node in the Solid Mechanics interface. A penalty factor formulation for the contact analysis has been used for the following contact pairs with assigned friction coefficient *μ* = 0.2: cage-shim pair, pad-shim pairs, and pad-shell pairs (see Fig. 6). All the components inside the cable stack were considered bonded after impregnation and shared nodes along with their interfaces.

**Figure 9:**
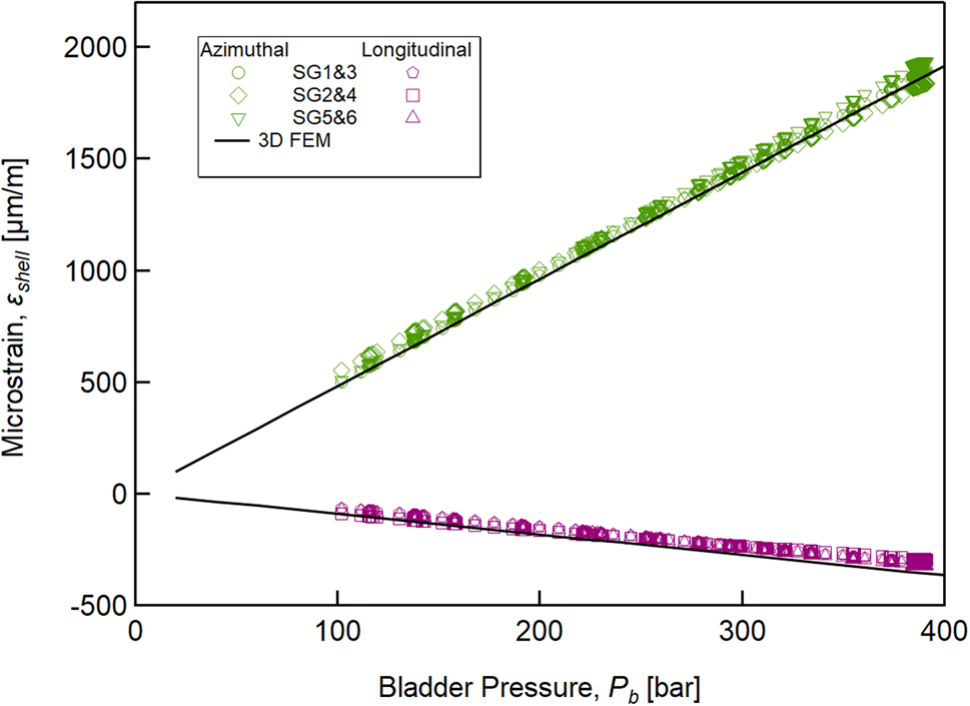
The azimuthal (green markers) and longitudinal (purple markers) strains measured at room temperature as a function of the pressure in the bladder (markers). The 3D FEM model (continuous black lines) reproduces the experimental linear behaviour with sufficient accuracy.

In order to validate the 3D model, we first measured the variation in strain recorded by the gauges as a function of the bladder pressure, *P*_*b*_. Gauge pairs SG1 and SG3, SG2 and SG4, and SG5 and SG6 did not measure exactly the same value due to expected not perfect symmetry of the stress applied on the aluminium shell by the pads. However, as a first approximation, it is the average value of the two that determines the pressure on the cable. Therefore, to mitigate any effects due to the imperfect geometry of the components and the final assembly, the values of the strain gauge pairs 1–3, 2–4, and 5–6 have been averaged. The averages thus obtained are shown in Fig. 9 (namely: SG1&3, SG2&4, and SG5&6). A linear relationship between the measured strain and the bladder pressure is found, up to *P*_*b*_ ≅ 500 bar. Figure 9 also shows the 3D simulation results, which are in excellent agreement with the experimental data. To verify the distribution of pressure on the cable’s surface, during these validation tests we placed pressure-sensitive films from FujiFilm Corporation (*Prescale* films) between the cable and the upper titanium pad. The tests with Prescale films showed no evidence of stress concentrations at room temperature. We do not expect any stress concentrations at 4.3 K, as the effects of the differential thermal contractions are fully isotropic.

**Figure 10:**
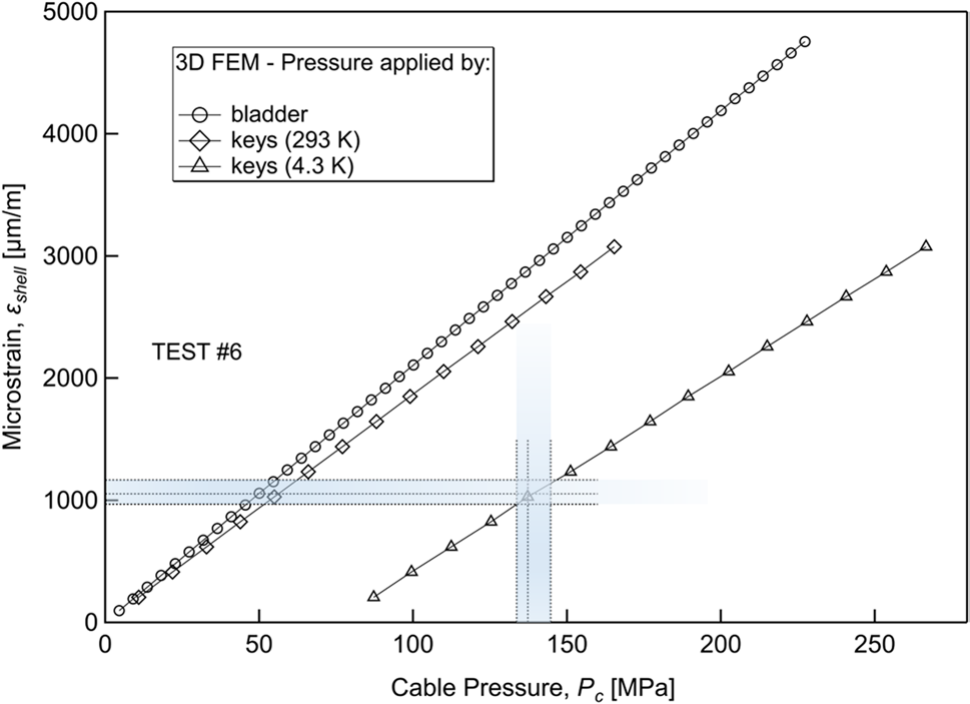
The relation between the azimuthal strain on the aluminium shell, evaluated at the positions depicted in Fig. 3, and the pressure on the 10-mm-wide cable calculated by the 3D FEM. The pressure was generated by a bladder (open circles) or by the interference between the keys and the Ti-6Al-4V components at 293 K (open diamonds) and at 4.3 K (open triangles). The procedure to assess the load on the cable is here resumed for Test #6. The measured strain averaged on the pair gauges SG1&3, SG2&4, and SG5&6 were 1050 μm/m, 970 μm/m, and 1160 μm/m, respectively. The pressure was estimated by using the highest measured strain (145 MPa), whereas the error was estimated as the difference between the maximum and minimum pressure values.

Finally, the transverse pressure applied on the cable at cryogenic temperature (4.3 K) by the Al shell (via the 316L keys and the Ti6Al4V components) was estimated with the 3D FEM model. At first, the correlation between the strain on the Al shell and the pressures on the cable at RT (*warm*) and 4.3 K (*cold*) was obtained from the FEM 3D model (see Fig. 10). Then, the measured strain values SG1&3, SG2&4, and SG5&6 were used to extrapolate the pressures on the cable (both warm and cold) by drawing a horizontal line corresponding to each of the aforementioned strain values, which intercepts the curves in the graph at the sought pressure value. As an example, the grey bands plotted in Fig. 10 illustrate the range of variability of strain values SG1&3, SG2&4, SG5&6 recorded in Test #6. The pressure values have been interpolated by using the three averaged values SG1&3, SG2&4, and SG5&6. As an estimate of the pressure acting on the cable, we chose the maximum obtained value of pressure: in Test #6, the maximum pressure is therefore 145 MPa, at a strain value of 1160 µm/m measured by SG5&6), with an error assessed by the difference between the maximum and minimum value (11 MPa).

Table 2 summarizes the transverse pressures (and their errors) on the cable at RT and 4.3 K calculated by using the different strain gauge values, for each load condition. The “*warm*” column in Table 2 represents the pressure on the cable, applied through to the bladders, to create the gap for the insertion of the stainless steel keys. For the high-pressure tests, the applied load should be lower than the final pressure obtained at cold, so that the full yielding of the cable (and possible filament cracks) occurs at 4.3 K. With the exception of the first test, this condition was always fulfilled; in the first test, the maximum pressure was anyhow too low for producing cable yielding and filaments cracks.

**Table 2:** Transversal pressure acting on the cable stack at RT and 4.3 K during the eleven different tests.

Test ID	Cable pressure, *P*_*c*_ [MPa]		Calculated interference [μm]	Variation of key heights [μm]
	By bladder 293 K	By interference 4.3 K		
1	100 ± 9	80 ± 3	0	0
2	115 ± 7	132 ± 4	118 ± 9	150 ± 29
3	*n.a*	76 ± 2	− 9 ± 5	0
4	130 ± 4	147 ± 13	152 ± 30	202 ± 28
5	118 ± 4	75 ± 3	− 11 ± 7	0
6	122 ± 4	145 ± 11	147 ± 25	202 ± 28
7	166 ± 8	184 ± 9	236 ± 21	304 ± 20
8	175 ± 14	76 ± 5	− 9 ± 12	0
9	167 ± 4	181 ± 7	229 ± 16	304 ± 20
10	181 ± 8	211 ± 12	297 ± 27	348 ± 30
11	215 ± 15	75 ± 2	− 11 ± 11	0

The pressure is estimated in 6 different location by using the strain measured on the Al shell at RT temperature after a thermo-cycle. The interference is calculated with a 3D FEM model at room temperature, whereas the variation of the key heights is evaluated experimentally.

Nominal interference was estimated by measuring the variation of the height of the keys with respect to reference values (Test # 1). First, the keys’ heights were determined as the average of three different measurements along its length by means of a Palmer micrometer. Then, for each specific test, the reference heights were subtracted to the corresponding heights, and the obtained values were finally averaged, to obtain the nominal interference. Table 2 also reports the effective interference values at RT between the keys and the lower pad (see Fig. 6, left) obtained by the FEM. These effective interferences differ from the nominal ones, presumably due to the copper plasticization occurring within the cable. In facts, since the FEM does not take into account the Cu yielding in the cable, the calculated interference was always lower than the variation of the height of the inserted keys. This simplification does not impact significantly the estimated pressure on the cable because the plasticity of copper only affects the pressure distribution on the cable (making it more homogeneous) and not its average value. The negative interference values obtained in subsequent unloading tests (Tests #3, #5, #8, and #11) and reported in Table 2 are entirely attributable to the plasticization of the copper generated by previous high-pressure tests: at RT the reference keys are not anymore in contact with the U-cage. It is also interesting to notice that the difference between real and simulated interference increases, as the applied pressure increases, because the plasticization of copper gradually becomes more important.

### ***I***_***c***_ measurements in the FReSCa test station

The FReSCa test station is the CERN facility for the acceptance tests of the superconducting cables for the LHC. The cable stack was inserted into FReSCa after each loading at the target pressure. The station allows tests up to a current of 32 kA in a background field up to 9.6 T at 4.3 K and 1.9 K. In this study, the PIT cable *I*_*c*_ were measured at a temperature of 4.3 K in a background field comprised in the range 4.4–9.6 T. The *V–I* characteristics were recorded independently on both sample legs by measuring the voltage drop with two sensitive digital nano-voltmeters (Keithley 2182A), and the current with direct current–current transformers. Additionally, a fast track HBM Gen3i data recorder system was used for monitoring the resistive transition and for quench analysis. The FReSCa dipole magnet was designed to ensure 95% field homogeneity along ~ 700 mm, which corresponds to the high-pressure length monitored by the voltage tap pair FM.

The background field was applied parallel to the large surface of the cable stack and perpendicular to the current, along the direction which maximizes the peak field, *B*_*p*_ (i.e., at low pressure, the peak field reaches values as high as 11.6 T at the background field of 9.6 T). We used the magnetic field interface^66^ provided by the COMSOL Multiphysics software to calculate the self-field map^65^.

**Figure 11:**
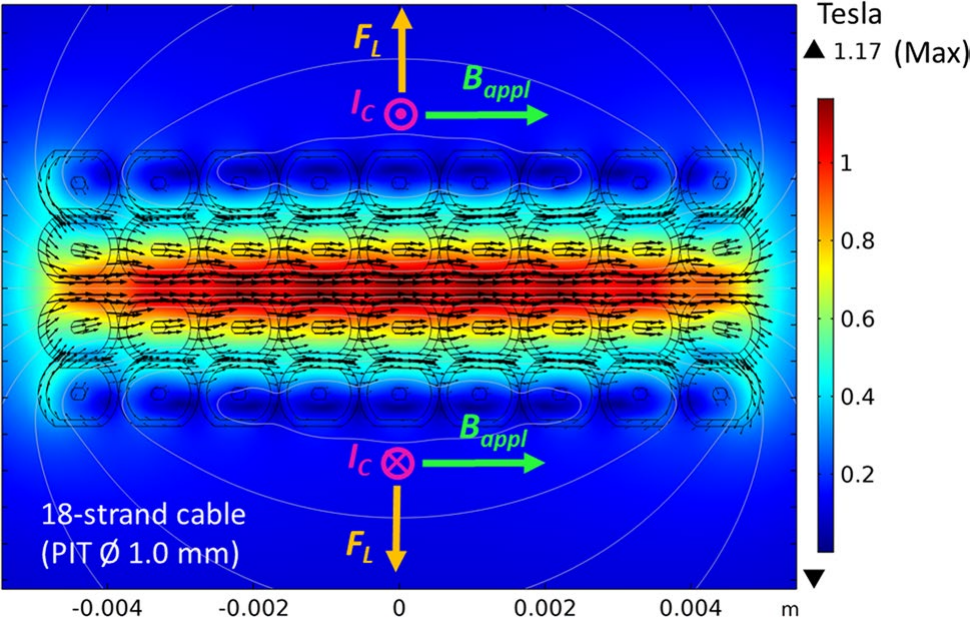
The self-field map calculated by finite elements software (COMSOL Multiphysics) at 10 kA. The peak field lies at the centre of the stack, with a negligible *y*-component. The white lines represents the contour plot (lines of constant magnitude) of the potential vector, directed along the *z*-direction. The background field is chosen in such a way to optimize the peak field. The resulting Lorentz forces, *F*_*L*_, acting on the current-carrying sample, are also depicted.

Due to the anti-parallel current flow, the peak field is located at the centre of the cable stack (Fig. 11), so the earliest quench is expected to starts from the central region of the sample. The directions of both the applied magnetic field and the Lorentz forces are also depicted in Fig. 11. The peak field was calculated as the sum of the applied field and the self-field produced by the current passing through the Rutherford cables. The self-field was estimated at 0.117 T/kA in our experimental setup.

Current ramps of 20, 40, and 300 A/s and voltage tap pairs with a distance of 69.5 cm (FM pair) were chosen for measuring the *V–I* characteristics. The different current ramp rates did not affect the quench currents; however, lower ramp rates made it possible to record more data points during the transition, thus allowing a more precise determination of the critical current. No significant differences were observed when the *I*_*c*_ data were analysed using different voltage tap pairs. This finding can be seen as further confirmation that the pressure, longitudinally, is uniformly distributed.

Due to the thermal runaway, it was not possible to determine the critical current according to the IEC61788-2 standard^67^: therefore, the recommended electrical field criterion (*E*_*c*_ = 0.1 μV/cm) was reduced to 3 × 10^–2^ μV/cm, which corresponds to ~ 2.085 μV at the FM voltage pair.

## Conclusions

The effect of a transverse load applied at cryogenic temperature on the critical current of a reacted and impregnated Nb_3_Sn Rutherford cable stack of PIT technology has been investigated.

No permanent critical current reduction was detectable below 130 MPa, as a full recovery of the initial value when the pressure was released was clearly observed.

At a peak field of 11.6 T, an irreversible reduction of *I*_*c*_ of less than 1.5% (8%) was observed after applying a load of 145 MPa (184 MPa). The irreversibility arises from the residual stresses generated by the plastic deformations of the copper stabilizer, which in turn induce permanent changes of the strain state within the Nb_3_Sn subelements.

The analysis of the *I*_*c*_ *vs*. *B*_*p*_ curves carried out using a simplified version of the ESE model has shown that this type of current reduction keeps the pre-factor constant *C* unchanged; any reduction of *I*_*c*_, whether reversible or not, is fully governed by the strain-induced variations of the upper critical field *B*_*c2*_. At higher pressures, estimated between 180 and 210 MPa, the prefactor *C* is significantly decreased, thus suggesting that a new mechanism for the permanent reduction of the *I*_*c*_ is taking place. At those pressures, it is indeed plausible to believe that cracking of filaments occurs, with detrimental consequences for the Nb_3_Sn cable’s electrical performances. It is worth pointing out that the boundary and threshold pressures reported in this study (130 MPa and 180–210 MPA, respectively) are specific to the heat treatment used, the wire layout and the design of this particular cable. These values can change substantially in other types of cables. Nonetheless, for state-of-the-art high-*J*_*c*_ wires we do not expect substantially higher values, and the proposed method retains its general validity.

The experimental results and the analysis methodology discussed in this paper can be extended to evaluate the stress tolerance of any Rutherford cable subjected to transverse pressure, as well as to discriminate the origin of the permanent reduction of the critical current, whether due to the plasticization of the copper stabilizer or the formation of cracks in the superconducting material.
